# Multi-Axis Functional Mechanisms of the Milpa Diet in Obesity: A Scoping Review

**DOI:** 10.3390/nu18121991

**Published:** 2026-06-19

**Authors:** Josué Ramos, Rogelio Salas, Carolina Salazar-Guerrero, Jimena Gaspar, Mirna E. Santos, Marcelo Hernández-Salazar, Silvia García, Marina Ródenas-Munar, Sofía Montemayor, Daniela Rodrigues, Cristina Bouzas, Josep A. Tur

**Affiliations:** 1 Population Nutrition Laboratory and Food Safety Control, Faculty of Public Health and Nutrition, Autonomous University of Nuevo León, 66455 San Nicolás de Los Garza, Nuevo León, Mexico; 2 Department of Nutrition, Faculty of Medicine, Autonomous University of Yucatán, 97000 Mérida, Yucatán, Mexico; 3 Research Group on Community Nutrition & Oxidative Stress, University of the Balearic Islands-IUNICS, 07122 Palma de Mallorca, Spain; 4 Centro de Investigación Biomédica en Red Fisiopatología de la Obesidad y la Nutrición (CIBEROBN), Institute of Health Carlos III, 28029 Madrid, Spain; 5 Health Research Institute of Balearic Islands (IdISBa), 07120 Palma de Mallorca, Spain; 6 Independent Researcher, 66455 San Nicolás de Los Garza, Nuevo León, Mexico; 7 Research Centre for Anthropology and Health, University of Coimbra, 3001-401 Coimbra, Portugal; drdc@uc.pt; 8 Department of Life Sciences, University of Coimbra, 3000-456 Coimbra, Portugal

**Keywords:** milpa diet, obesity, bioactive compounds, inflammation, oxidative stress, gut microbiota, metabolic health

## Abstract

**Background:** Obesity is a multifactorial metabolic disorder characterized by chronic low-grade inflammation, oxidative stress, mitochondrial dysfunction, lipotoxicity, dysregulated adipogenesis, and alterations in the gut microbiota, which collectively contribute to insulin resistance and cardiometabolic complications. In this context, dietary patterns rich in bioactive compounds have gained relevance as potential strategies to modulate these interconnected pathways. **Objective:** To assess the potential of the Milpa Diet (a sustainable, plant-dominant Mesoamerican eating pattern centered on the ancient three sisters’ polyculture of maize, beans, and squash, along with chili) as a culturally relevant, multi-axis functional dietary pattern, and to evaluate the molecular mechanisms underlying obesity-associated with metabolic dysfunction. **Methods:** A scoping review of preclinical and clinical studies was conducted using Medline via PubMed, Scopus, and Web of Science databases. The ChEMBL database was also used to identify chemical structures. The search focused on evidence related to inflammation, oxidative stress, adipogenesis, lipotoxicity, mitochondrial function, and gut microbiota modulation in the context of the main foods of the Milpa Diet, including maize, legumes, chili peppers, nopal, and quelites. Studies were selected based on peer-review status and their relevance to molecular, metabolic, and functional outcomes. **Results**: The current evidence shows that the core components of the Milpa Diet provide dietary fiber and a broad range of bioactive compounds, such as flavonoids, carotenoids, capsaicinoids, phenolic acids, pigments, and vitamins, which exhibit antioxidant and anti-inflammatory effects. These compounds have been associated with modulation of adipogenesis and lipotoxicity, preservation of mitochondrial function, and favorable regulation of gut microbiota composition and activity, collectively influencing metabolic pathways relevant to obesity. **Conclusions:** Overall, mechanistic and emerging clinical evidence suggests that the Milpa Diet represents a multi-axis nutritional strategy with potential to mitigate obesity-related metabolic dysfunction through coordinated effects on inflammation, oxidative stress, adipogenesis, lipotoxicity, mitochondrial function, and gut microbiota regulation. Although comprehensive clinical trials evaluating this dietary pattern as an integrated intervention remain limited, current evidence supports its relevance for future translational research, public health strategies, and the development of sustainable dietary models aimed at improving metabolic health.

## 1. Introduction

Obesity is a chronic pathology characterized by an excessive accumulation of adipose tissue that compromises the structural and functional integrity of the organism, thereby increasing the risk of premature mortality [1,2]. Clinically, obesity is defined by a body mass index (BMI) > 30 kg/m^2^; however, accurate diagnosis demands measuring waist circumference (>102 cm in men; >88 cm in women) to identify central adiposity and underlying cardiometabolic risk [3,4]. At the public health level, the World Health Organization (WHO) reports that more than one billion individuals were affected in 2024, linking this crisis to one in every eight deaths worldwide [5]. In Mexico, the 2023 ENSANUT (Encuesta Nacional de Saludy Nutrición) reported a prevalence of 76.2% among adults, with associated mortality primarily attributed to diabetes mellitus (64.4%), nephropathies, and ischemic heart disease [6]. The etiology of obesity is multifactorial, with 40–70% heritability, which is further amplified by prenatal factors and obesogenic environments, including physical inactivity and consumption of ultra-processed foods [4,7]. Systemically, visceral adiposity functions as the driving force behind insulin resistance, hypertension, and hepatic steatosis. Moreover, the chronic pro-inflammatory state increases the risk of osteoarthritis, obstructive sleep apnea, and neoplasms of the colon, breast, and pancreas [8,9].

The Western diet saturates adipose tissue, leading to ectopic fat accumulation in which diacylglycerols (DAGs) and ceramides interfere with insulin signaling, while lipopolysaccharide (LPS) translocation activates endoplasmic reticulum stress through the Inositol-Requiring Enzyme 1—X-box binding protein 1 (IRE1–XBP1) pathway, thereby promoting hepatic gluconeogenesis [10,11]. At the systemic level, obesity induces mitochondrial dysfunction that increases reactive radical oxygen species (ROS) production through Nicotinamide adenine dinucleotide phosphate (NADPH) oxidase activity, damaging deoxyribonucleic acid (DNA) and reducing nitric oxide bioavailability [12]. This redox injury is associated with macrophage infiltration that activates the nucleotide-oligomerization domain (NOD)-like receptor protein 3 (NLRP3) inflammasome and the nuclear factor kappa B (NF-κB) pathway, leading to the secretion of cytokines such as tumor necrosis factor-alpha (TNF-α) and interleukin-6 (IL-6) that phosphorylate Insulin Receptor Substrate 1 (IRS-1), thereby consolidating insulin resistance [13,14]. Simultaneously, the excess of free fatty acids induces adipocyte pyroptosis and restricts functional adipogenesis through the inhibition of Peroxisome Proliferator-Activated Receptor Gamma (PPARγ) and CCAAT/enhancer-binding protein alpha (C/EBPα), promoting cellular hypertrophy and impairments in perilipin function [15,16]. This scenario is exacerbated by impaired mitochondrial biogenesis and a reduction in electron transport chain activity (OXPHOS), together with diminished Krebs cycle activity, collectively enforcing a state of metabolic inflexibility toward anaerobic glycolysis [17,18]. Finally, gut dysbiosis characterized by a high *Bacillota/Bacteroidota* ratio and the loss of *Akkermansia muciniphila* decreases the synthesis of short-chain fatty acids (SCFAs), which weakens G protein-coupled receptors 41 and 43 (GPR41/43) signaling and increases systemic inflammation through Toll-like Receptor 4 (TLR4) activation, thereby perpetuating the cycle of dysfunctional adipogenesis [19,20].

The aim of this study was to assess the potential of the Milpa Diet (a sustainable, plant-dominant Mesoamerican eating pattern centered on the ancient three sisters’ polyculture of maize, beans, and squash, along with chili) as a culturally relevant multi-axis functional dietary pattern, and to evaluate the molecular mechanisms underlying obesity-associated metabolic dysfunction.

## 2. Methods

This scoping review was conducted using a structured and reproducible search strategy aimed at integrating nutritional, molecular, and experimental evidence related to the Milpa Diet and its role in obesity and associated metabolic dysfunction. Data collection was carried out across widely recognized scientific databases: Medline via PubMed, Scopus, and Web of Science. Additionally, the ChEMBL database was also used as a curated source for the identification and verification of chemical structures and biochemical annotations of bioactive compounds present in characteristic foods of the Milpa system. No strict limits were set on publication year in order to include both recent studies and foundational research relevant to the understanding of the mechanisms analyzed; however, priority was given to literature published within the last five years, while earlier studies were incorporated when they provided essential mechanistic, analytical, or conceptual evidence not available in more recent publications.

The search strategy was developed in three complementary phases:

(1) First phase: The search initially focused on foods characteristic of the Milpa Diet, including maize, beans, squash, chili, tomato, nopal, and quelites, with the aim of identifying studies describing their general nutritional composition, the presence of bioactive compounds, and their association with obesity and metabolic dysfunction. This stage prioritized evidence related to the six functional axes: inflammation, oxidative stress, adipogenesis, lipotoxicity, mitochondrial dysfunction, and gut microbiota modulation.

(2) Second phase: A more in-depth analysis was conducted on the molecular mechanisms associated with specific bioactive compounds derived from milpa foods. Approximately 20 representative compounds were selected, including flavonoids (anthocyanins), capsaicinoids, carotenoids, phenolic acids, pigments, and vitamins, in order to identify cellular signaling pathways, molecular targets, and metabolic effects linked to obesity, inflammation, oxidative stress, adipogenesis, lipotoxicity, mitochondrial function, and gut microbiota regulation. The chemical structures and biochemical annotations of these compounds were verified using the ChEMBL database.

(3) Third phase: The search focused on experimental studies, both in vitro and in vivo, evaluating the effects of Milpa Diet foods and their bioactive compounds in obesity models. Human studies were prioritized when available, while animal studies—primarily in rodent models, were included when they provided relevant mechanistic insights for the interpretation of observed effects. The identification of relevant studies was optimized through the use of Medical Subject Headings (MeSH) descriptors and Boolean operators, combining terms related to obesity, and metabolic dysfunction with those corresponding to characteristic foods and bioactive compounds of the Milpa Diet.

MeSH terms used were: “Milpa Diet”, “Traditional Mexican Diet”, “Bioactive compounds”, “Obesity”, “Oxidative stress”, “Inflammation”, “Antioxidant”, “Anti-inflammatory”, “Gut microbiota”, “metabolic dysfunction”, “insulin resistance”, “Mitochondrial dysfunction”, “Adipogenesis”, “Lipotoxicity”, and “In vitro studies”, as well as “maize”, “corn”, “bean”, “Phaseolus vulgaris” “squash”, “pumpkin”, “Cucurbita pepo”, “chili”, “Capsicum annuum”, “tomato”, “Solanum lycopersicum”, “nopal”, “Opuntia ficus-indica”, “quelites”, “squash”, “polyphenols”, “flavonoids”, “carotenoids”, “capsaicinoids”, “phenolic acids”, “betalains”, and “tocopherols”.

The complete search strategies for each database, including the search strings and the exact Boolean operators used, filters applied, and number of records reported are shown in Supplementary Table S1.

Inclusion criteria were limited to peer-reviewed articles published in English or Spanish that reported preclinical or clinical outcomes with molecular, metabolic, or functional relevance. Non-systematic reviews, gray literature, and studies with significant methodological limitations were excluded.

In total, 344 articles were identified with the detailed search criteria mentioned above. The following search filters were applied: studies in animals (rats and mice), studies conducted in humans, clinical trials, studies conducted in adults, and studies conducted in children (Controlled Clinical Trial or Randomized Clinical Trial).

This article was initially conceived as a comprehensive review and not as a systematic review. However, the high number of references included in the manuscript made it necessary to present the literature selection process in a clearer and more transparent manner. Therefore, a flowchart based on the Preferred Reporting Items for Systematic Reviews and Meta-Analyses (PRISMA) guidelines was added as a complementary reporting tool, with the purpose of summarizing the identification, eligibility assessment, exclusion and final inclusion of the studies considered for the review.

**Figure 1:**
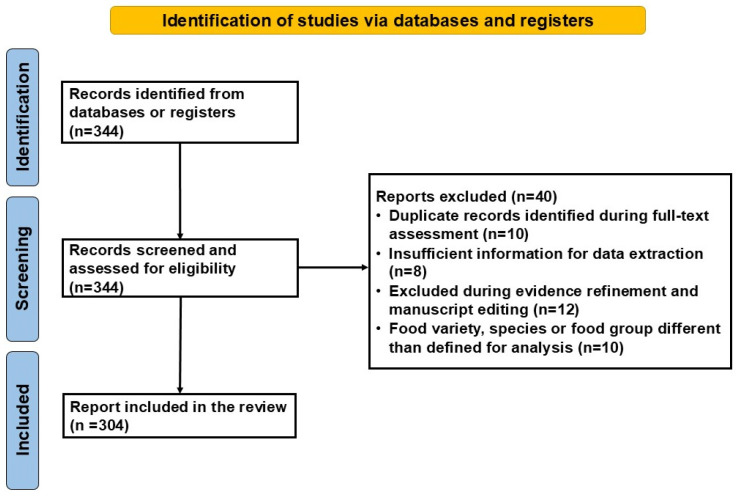
Flowchart of the study.

The search was standardized and conducted by two reviewers in May 2026, working in parallel, who initially screened titles and abstracts, and subsequently reviewed full-text articles. Disagreements were first solved by discussion and consensus between the two reviewers. Discrepancies were resolved through agreement with a third reviewer. The search was supplemented by reviewing the bibliographic references included in each retrieved article. If an original article was found, it was included in the review. Accordingly, 344 records were initially identified and evaluated. After full-text evaluation and the editorial screening process, 40 reports were excluded for specific reasons: duplicate records identified during full-text evaluation (*n* = 10), insufficient information for data extraction (*n* = 8), exclusion during evidence refinement and manuscript editing (*n* = 12), and differences in variety, species or food group with respect to previously defined analytical criteria (*n* = 10). Finally, 304 studies were included in this review, and the selected evidence was synthesized and organized according to the six previously defined functional axes to evaluate the potential of the Milpa Diet as a multiaxial dietary pattern in the context of obesity. The process used to identify and select articles is shown as a flowchart in Figure 1.

## 3. Overview of the Milpa Diet

**Figure 2:**
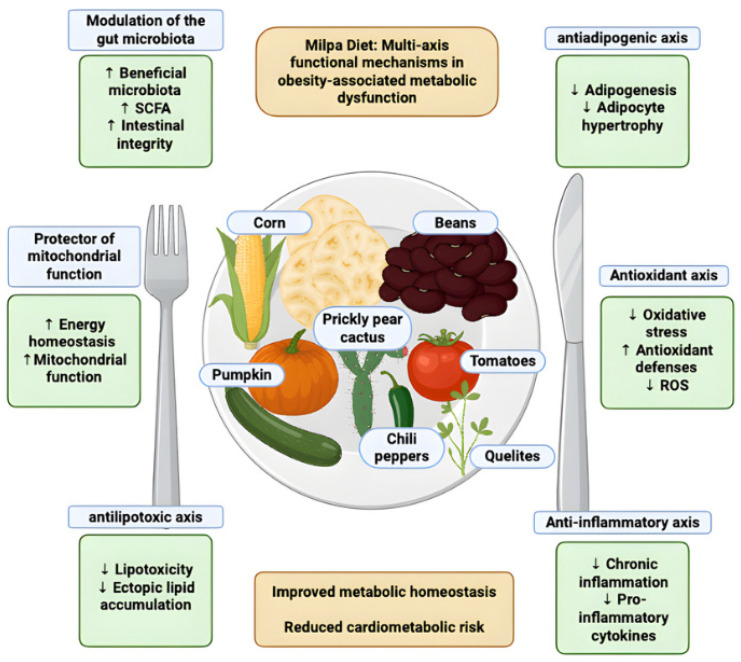
Multi-axis integrative scheme of the Milpa Diet in obesity. The figure summarizes the main functional mechanisms through which the core foods of the Milpa Diet—maize/corn, beans, squash, chili peppers, tomatoes, prickly pear cactus, and quelites—may contribute to the modulation of obesity-associated metabolic dysfunction. These mechanisms include antioxidant and anti-inflammatory effects, anti-adipogenic and anti-lipotoxic actions, protection of mitochondrial function, and modulation of the gut microbiota, ultimately converging in improved metabolic homeostasis and reduced cardiometabolic risk. Arrow pointing up: Increase; Arrow pointing down: Decrease.

Dietary natural compounds are an adjuvant therapeutic strategy for obesity due to their ability to mitigate oxidative damage and associated pathologies. Their mechanism of action encompasses antioxidant and anti-inflammatory properties, including the neutralization of free radicals, modulation of the gut microbiota, and regulation of gene transcription factors. Among the most relevant bioactive groups are polyphenols, carotenoids, capsaicinoids, isothiocyanates, and catechins [21]. Certain dietary patterns transcend basic nutritional provision by providing functional and therapeutic properties. An example is the Milpa Diet (Figure 2), recognized by the Food and Agriculture Organization of the United Nations as an “Important Agricultural Heritage System”. This traditional polyculture is a dynamic ecosystem for genetic conservation, in which species such as maize (*Zea mays* L.), bean (*Phaseolus vulgaris* L.), squash (*Cucurbita pepo* L.), chili pepper (*Capsicum annuum* L.), tomato (*Solanum lycopersicum* L.), nopal (*Opuntia ficus-indica*), and quelites stand out [22]. This model is notable for its nutritional complementarity: maize provides energy and complex carbohydrates; beans serve as a source of plant proteins and micronutrients; squash supplies fiber, vitamins, and minerals; and chili peppers contribute additional bioactive compounds. The synergy among these crops enables comprehensive coverage of nutritional requirements, with the exception of vitamin B_12_ [23,24]. Taken together, these components increase energy homeostasis and metabolic flexibility.

In addition, the Milpa Diet regularly incorporates avocado, pumpkin seeds (*Cucurbita pepo* L.), amaranth (*Amaranthus* spp.), cacao (*Theobroma cacao*), and chia (*Salvia hispanica*). It also includes fruits such as soursop (*Annona muricata* L.) and prickly pear (*Opuntia ficus-indica* L.), along with sources of resistant starch such as sweet potato (*Ipomoea batatas*). Animal protein sources include fish, seafood, dairy products, and edible insects such as “chapulines”. These foods provide a complex matrix of bioactive compounds, including polyphenols, dietary fiber, unsaturated fatty acids, and essential micronutrients. The synergy of these components exerts modulatory effects on the molecular pathophysiology of obesity, influencing inflammation and metabolic regulation.

Avocado (*Persea americana*) is characterized by a matrix rich in oleic acid (>70%), dietary fiber, and bioactive compounds such as phytosterols, carotenoids, and polyhydroxylated fatty alcohols (avocadenol and avocadin). These components act synergistically to mitigate oxidative stress and systemic inflammation by contributing to mitochondrial function and modulating lipid metabolism [25,26]. Clinical evidence associates its regular consumption with a potential benefit on visceral adipose tissue and body fat distribution, particularly in women with obesity [25]. Likewise, it promotes a reduction in small, dense low-density lipoprotein (LDL) particles and oxidized LDL, thereby attenuating cardiometabolic risk [27]. At the intestinal level, avocado increases microbial diversity and the production of SCFAs, promoting energy homeostasis and regulating systemic inflammation [28,29].

Pumpkin seed (*Cucurbita pepo*) is notable for its high protein density (30–35%) and a lipid profile rich in linoleic acid and γ-tocopherol. Its bioactive fraction includes phytosterols (β-sitosterol), phenolic compounds such as (–)-epicatechin, and specific polysaccharides such as PPS3, which exert antioxidant effects and modulate lipid metabolism [30,31,32]. In the context of obesity, these components modulate systemic inflammation and optimize the lipid profile [33]. At the molecular level, the polysaccharides of *C. pepo* activate the AMPK pathway, promoting fatty acid oxidation and inhibiting hepatic lipogenesis. Likewise, inhibition of α-amylase and α-glucosidase has been reported, which supports postprandial glycemic control [34,35]. Recent evidence suggests that these mechanisms directly reduce body weight and adiposity, without altering caloric intake, through positive modulation of the gut microbiota [32].

Amaranth (*Amaranthus* spp.) is a pseudocereal of high nutritional and functional value, with increasing relevance in the dietary management of obesity. It is distinguished by its high-biological-value protein (rich in lysine and arginine), complex carbohydrates, and a lipid fraction dominated by squalene, making it one of the richest plant sources of this compound. Its bioactive matrix includes tocopherols, betalains, and peptides with antihypertensive and anti-inflammatory properties [36,37,38]. In the pathophysiology of obesity, these compounds provide protection against oxidative damage and modulate energy metabolism. In particular, amaranth bioactives have been shown to regulate the PI3K/AKT pathway, which controls adipogenesis and insulin sensitivity [39]. Although clinical evidence is emerging, its capacity to enhance energy homeostasis suggests that regular amaranth consumption promotes adiposity reduction and optimizes body composition within functional nutritional strategies [36,37,38,39].

Cacao (*Theobroma cacao*) is nutritionally a complex matrix of fiber, lipids, and minerals, notable for its high concentration of flavanols (particularly (–)-epicatechin and procyanidins B2 and C1) and methylxanthines such as theobromine. It is important to note that post-harvest processing, particularly fermentation, can reduce the density of these bioactive compounds [40,41]. In the pathophysiology of obesity, procyanidin oligomers have demonstrated superior bioactivity in preventing fat mass gain and insulin resistance induced by high-fat diets [42]. Epicatechin improves endothelial function and attenuates chronic inflammation, while theobromine improves lipid and glucose metabolism [43,44]. Preclinical evidence confirmed that the synergistic action of these compounds reduced body weight, triglycerides, and LDL cholesterol, establishing cocoa as a key functional agent [45,46].

Chia (*Salvia hispanica*) is notable for its lipid density (20–34%), with predominance of α-linolenic acid (ALA, omega-3, =60% of total lipids) and linoleic acid. It possesses a complete protein profile (16–26%) and a high dietary fiber content (up to 41%), primarily insoluble, which promotes satiety and intestinal motility [47,48]. Its bioactive fraction includes phenolic acids (chlorogenic and caffeic) and flavonoids such as quercetin and kaempferol [49]. In the pathophysiology of obesity, omega-3 fatty acids and antioxidants from chia regulate critical molecular pathways such as AMP-activated protein kinase (AMPK), Mitogen-Activated Protein Kinase (MAPK), and NF-κB y PPAR-γ, mitigating oxidative stress and adipogenesis [50]. Furthermore, a positive modulation of the gut microbiota was observed, linked to a reduction in inflammatory markers [51]. Although animal models reported significant decreases in BMI and fat mass, clinical evidence in humans is still limited and does not show consistent anthropometric effects, which necessitates further validation in controlled studies [51,52].

Soursop (*Annona muricata* L.) has a dense profile of antioxidant vitamins (A, C, E), minerals (Mg, K), and a dietary fiber fraction with high free radical scavenging capacity [53]. Its phytochemical matrix integrates flavonoids such as quercetin and luteolin, as well as carotenoids and tocopherols distributed throughout the pulp and leaves [54,55]. In the functional management of obesity, the components of *A. muricata* exert hypoglycemic and hypolipidemic effects that are critical for mitigating systemic inflammation. Its dietary fiber promotes satiety and gut microbiota modulation, while its polyphenols optimize insulin sensitivity and attenuate ectopic lipid accumulation [53,54,55]. These combined actions promote body weight regulation and the preservation of lean mass relative to adipose tissue [54].

The prickly pear (*Opuntia ficus-indica* L.) has fiber, vitamin C, and a lipid profile rich in linoleic and oleic acids. Its bioactive matrix is unique for the presence of betalains (betacyanins and betaxanthins) and specific metabolites such as isorhamnetin, as well as eucomic and piscidic acids, with the latter being basic in the regulation of cellular metabolism [56,57,58]. At the molecular level, these compounds inhibit triglyceride accumulation in adipocytes by interfering with adipogenesis and lipogenesis pathways. Piscidic acid acts on the Nuclear factor erythroid 2-related factor 2 (Nrf-2) and NF-κB pathways, increasing the antioxidant response and decreasing the secretion of pro-inflammatory cytokines such as IL-6 and interleukin-8 (IL-8) [59,60]. The prickly pear enhances a positive modulation of the gut microbiota, reducing taxa associated with metabolic endotoxemia while improving glucose tolerance and hepatic function [61,62]. While preclinical evidence is weight and fat mass loss, clinical translation to humans still requires larger-scale controlled trials to confirm its anthropometric efficacy [60,62].

Sweet potato (*Ipomoea batatas*) stands out for its high density of bioactive compounds, primarily anthocyanins in purple varieties and β-carotene in orange genotypes, along with caffeoylquinic acids [63,64]. These compounds exert potent antioxidant and anti-inflammatory activity through the activation of the Nrf2 pathway and the inhibition of NF-κB, thereby mitigating systemic oxidative stress [65,66]. Its fiber fraction and non-starch polysaccharides modulate the gut microbiota and promote satiety [67]. Preclinical evidence shows that sweet potato reduces adiposity and inhibits hepatic lipogenesis, thereby improving insulin sensitivity [68]. In clinical interventions, the consumption of purple varieties has demonstrated efficacy in reducing abdominal circumference and improving the lipid profile, reinforcing their functional value in obesity management [65,67].

Animal proteins derived from the marine sector offer functional relevance through their contribution of eicosapentaenoic acid (EPA) and docosahexaenoic acid (DHA), vitamins (D, B_12_), and peptides generated from protein hydrolysis. These peptides exhibit anti-obesogenic effects by modulating satiety-related hormones such as leptin and regulating postprandial glycemia [69,70]. Omega-3 fatty acids, particularly in phospholipid form, increase circulating adiponectin and attenuate systemic inflammation [71]. At the systemic level, the intake of marine proteins contributes to gut microbiota composition by limiting pro-inflammatory taxa and improving overall energy metabolism [72,73]. Although preclinical evidence confirms reductions in abdominal circumference and body weight, further clinical validation is required to solidify these recommendations within the comprehensive treatment of human obesity [69,74].

The Milpa grasshopper (*S. purpurascens*) stands out as a functional food due to its hydroacetic extracts (HA-A) and bioactive peptides derived from its protein hydrolysis. These components exhibit the highest antioxidant capacity (Oxygen Radical Absorbance Capacity -ORAC-, Trolox Equivalent Antioxidant Capacity -TEAC-, and 2,2-diphenyl-1-picrylhydrazyl -DPPH- assays), which is essential for mitigating oxidative stress and insulin resistance [75]. At the molecular level, the HA-A extract inhibits the cyclooxygenase-2 (COX-2) enzyme by up to 60%, thereby attenuating low-grade systemic inflammation. It also exhibits potent metabolic regulation by inhibiting α-glucosidase (73.8%) and pancreatic lipase (46.7%), mechanisms that delay glucose absorption and reduce the hydrolysis of dietary triglycerides, consequently lowering net caloric intake. Chemical characterization identified 13 key compounds, including phenolic acids and flavonoids, which—together with the peptides released through hydrolysis—confer to the grasshopper significant antidiabetic and anti-obesogenic properties with high functional impact [75].

**Figure 3:**
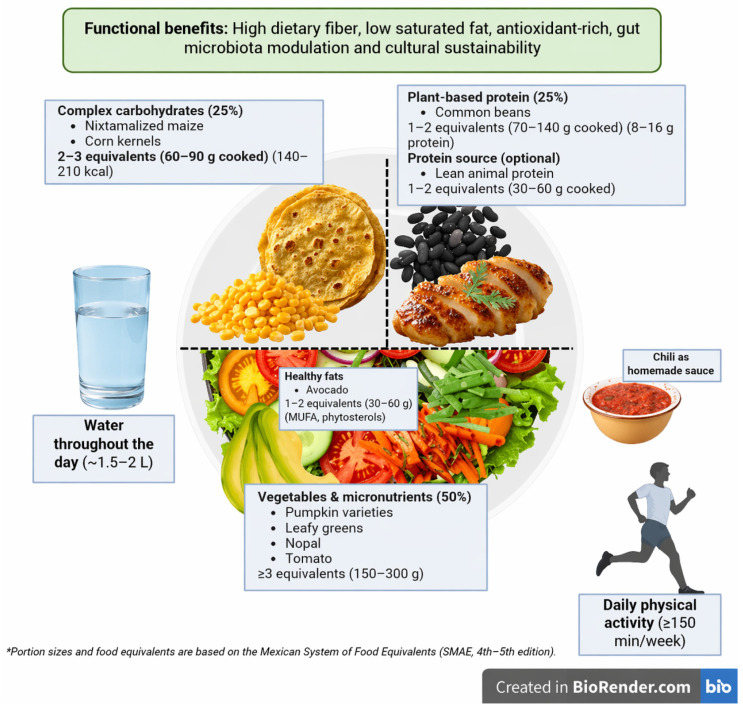
Example of a nutritionally balanced plate based on the Milpa Diet. The plate illustrates the distribution of complex carbohydrates (nixtamalized maize), plant-based protein (common beans), vegetables rich in micronutrients and bioactive compounds, and healthy fats (avocado). This dietary pattern provides adequate energy, fiber, vitamins, minerals, and antioxidants, while being culturally representative, affordable, and easy to assemble using locally available foods.

The Milpa system provides an integrated matrix of essential micronutrients, featuring an extensive vitamin spectrum (A, C, E, K, and B-complex) and critical minerals such as potassium, magnesium, iron, and zinc. This synergy ensures the protein, lipid, and carbohydrate balance required for cellular homeostasis and the fulfillment of daily dietary requirements [35]. This dietary model stands out for its density of bioactive compounds (polyphenols, phytosterols, saponins, peptides, and polyunsaturated fatty acids -PUFAs-). These metabolites neutralize free radicals by modulating antioxidant enzymes and reducing pro-inflammatory cytokines. Likewise, they regulate lipid and intestinal metabolism by inhibiting adipogenesis and reducing the luminal absorption of glucose and triglycerides. This protective action prevents metabolic and cardiovascular disorders, optimizing energy regulation and immune response [35]. The design of a balanced plate following the milpa dietary pattern is based on the synergistic combination of nixtamalized maize, beans, squash, and chili, as shown in Figure 3. This triad ensures optimal protein complementarity by balancing the profile of essential amino acids, while also providing complex carbohydrates, fiber, and a predominantly unsaturated lipid profile [76,77].

According to the literature, the structure should prioritize whole grains and legumes, complemented by an abundance of local vegetables and the moderate use of plant-based fats such as avocado or seeds [78,79]. A practical example integrates tortillas, beans, and squash, accompanied by guacamole and fresh salsas, which maximizes the bioavailability of micronutrients such as folates, calcium, and iron [80]. By excluding ultra-processed foods and prioritizing biocultural diversity, this model promotes food sustainability and cardiometabolic health in Hispanic–Latino populations [76,77].

Studies in Mexican adults have shown that the traditional dietary pattern, based on the milpa system and a low intake of ultra-processed foods, is associated with a lower prevalence of overweight and obesity, independently of sociodemographic factors or physical activity [81]. In school-aged populations, this dietary pattern acts as a protective factor, in contrast to the rise in obesity associated with modern diets rich in saturated fats and industrialized products [82]. The specific inclusion of nopal, beans, and avocado has been shown to reduce BMI and improve metabolic parameters in individuals with obesity [83]. Despite these benefits, systematic reviews indicate that the evidence remains heterogeneous due to variability in how adherence to this dietary pattern is measured [84]. Nevertheless, scientific consensus supports that the Milpa Diet, when preserved in its ancestral form and not displaced by ultra-processed foods, represents an effective strategy for the prevention of obesity and its comorbidities in Mexico [81,85].

## 4. Main Foods of the Milpa Diet

**Figure 4:**
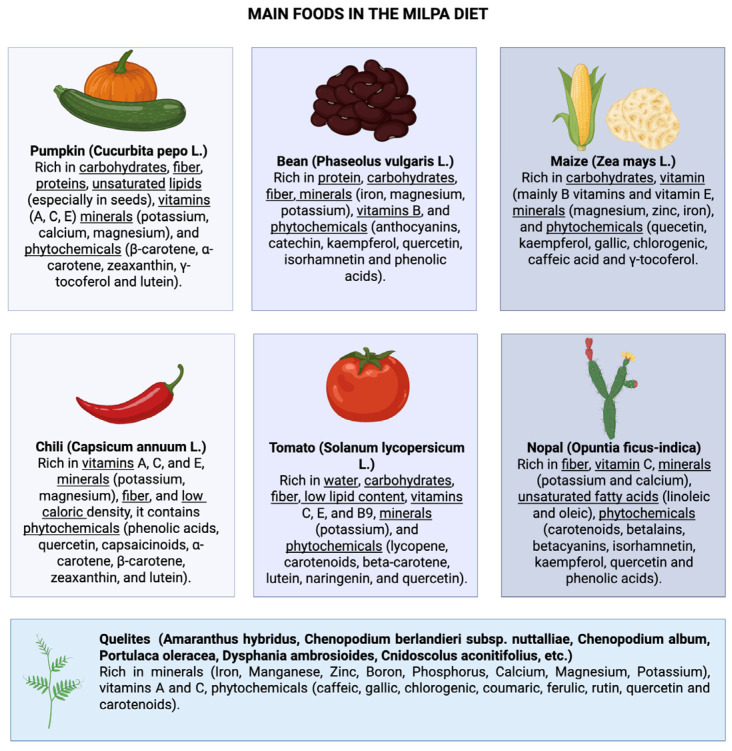
Representative foods of the Milpa Diet, including squash (*Cucurbita pepo* L.), beans (*Phaseolus vulgaris*), maize (*Zea mays*), chili pepper (*Capsicum annuum*), tomato (*Solanum lycopersicum*), nopal cactus (*Opuntia ficus-indica*), and quelites. These foods are rich in dietary fiber, complex carbohydrates, vitamins, minerals, and bioactive compounds such as phenolic acids, flavonoids, carotenoids, capsaicinoids, and betalains, which contribute to their potential health-promoting effects.

The primary foods comprising the Milpa Diet exhibit a remarkable profile of bioactive compounds with pleiotropic effects, capable of acting in a coordinated manner across multiple metabolic and cellular pathways relevant to obesity, as shown in Figure 4.

Maize (*Zea mays* L.), particularly pigmented varieties, represents a critical source of cyanidin-3-glucoside and phenolic acids (ferulic and p-coumaric acids), which, in synergy with its resistant starch and fiber content, exert multiaxial biological activity [86,87]. Its antioxidant and anti-inflammatory properties are based on the neutralization of reactive species and inhibition of the NF-κB pathway, thereby mitigating chronic low-grade systemic inflammation [88]. Its extracts exert potent anti-adipogenic and anti-lipotoxic effects by repressing transcription factors such as SREBP1 and key enzymes (ACACA, FASN), preventing preadipocyte differentiation and ectopic lipid accumulation [89]. At the cellular level, maize acts as a protector of mitochondrial function through activation of SIRT1 and AMPK pathways, enhancing fatty acid β-oxidation and energy homeostasis under metabolic stress [90]. Its complex carbohydrate fraction serves as a robust modulator of gut microbiota, promoting the production of short-chain fatty acids (SCFAs) and reducing dysbiosis, which translates into high insulin sensitivity and reduced adipose mass gain [88,91].

Similarly, common bean (*Phaseolus vulgaris* L.), particularly black and pinto varieties, constitutes a dense matrix of anthocyanins (delphinidin, malvidin, and petunidin) and flavonoids such as isorhamnetin, whose interaction with resistant starch and high-quality proteins defines its therapeutic potential [92,93]. This legume exerts strong antioxidant and anti-inflammatory effects by neutralizing reactive species and suppressing critical mediators such as COX-2, Inducible Nitric Oxide Synthase (iNOS), and pro-inflammatory cytokines (TNF-α, IL-1β), thereby mitigating chronic low-grade inflammation [94,95]. At tissue level, beans act as anti-adipogenic and anti-lipotoxic agents, reducing visceral fat and adipocyte size through repression of lipogenic genes and decreased circulating triglycerides, thereby protecting hepatic integrity [96]. Their role as mitochondrial protectors is indirectly evidenced by increased activity of carnitine palmitoyl transferase 1 (CPT1), enhancing lipid β-oxidation and cellular energy efficiency [97]. Their fiber and polyphenol content plays a key role in modulating gut microbiota, promoting the growth of beneficial taxa (*Bifidobacterium*, *Roseburia*) and SCFA production, reinforcing their anti-obesogenic effect without altering feed efficiency [95,98].

Squash (*Cucurbita pepo* L.) exhibits a phytochemical profile mainly composed of carotenoids such as β-carotene, α-carotene, lutein, and zeaxanthin, which, together with tocopherols and polyphenols, exert high antioxidant and anti-inflammatory effects. These compounds neutralize reactive oxygen species and modulate signaling pathways that mitigate chronic inflammation associated with obesity [99,100]. At metabolic level, the fruit acts as an anti-adipogenic agent by inhibiting key enzymes such as α-glucosidase and α-amylase, thereby limiting substrate availability for lipid accumulation in peripheral tissues [34]. Unsaturated fatty acids and sterols from its seeds promote lipid homeostasis, while compounds such as squalene and γ-tocopherol act as mitochondrial protectors, preserving membrane integrity under metabolic overload [30,101]. The polysaccharide PPS3 from squash stands out as a modulator of the gut microbiota, reversing high-fat diet-induced dysbiosis by increasing beneficial genera such as *Akkermansia* and *Parabacteroides*, and reducing the *Firmicutes/Bacteroidetes* ratio [32]. This restructuring of the gut ecosystem correlates with reduced body weight and adiposity, as well as improved lipid profiles and glucose tolerance making it potentially beneficial in obesity management [32,101].

Chili pepper (*Capsicum annuum* L.) is characterized by a dense matrix of carotenoids (lutein, zeaxanthin, β-carotene, and α-carotene) and capsaicinoids, primarily capsaicin, whose concentration varies by genotype [102,103]. This phytochemical combination, along with polyphenols such as quercetin, confers high antioxidant and anti-inflammatory ability by neutralizing reactive species and suppressing pro-inflammatory cytokines (TNF-α, IL-6) via modulation of TLR4 signaling [104,105]. Chili also demonstrates anti-adipogenic and anti-lipotoxic actions by inhibiting preadipocyte differentiation, reducing pancreatic lipase activity, and promoting fatty acid oxidation [106,107]. Its role as mitochondrial protector is linked to thermogenesis induction and energy expenditure, facilitating reductions in fat mass and abdominal circumference [104,108]. Chili is a potent modulator of gut microbiota, reducing the abundance of lipopolysaccharide (LPS)-producing Gram-negative bacteria (*Proteobacteria*). This structural shift in the microbiome, along with increased expression of tight junction proteins (ZO-1, occludin), strengthens intestinal barrier integrity, reducing metabolic endotoxemia and improving glucose and lipid metabolism [104,108].

Tomato (*Solanum lycopersicum* L.) stands out as a primary source of lycopene, a carotenoid with high antioxidant capacity, which, together with β-carotene, naringenin, and quercetin, forms a robust bioactive matrix [109,110]. Its antioxidant and anti-inflammatory effects are mediated through neutralization of reactive species and inhibition of NF-κB and AMPK pathways, reducing the pro-inflammatory cytokines (TNF-α, IL-6) expression and attenuating adipose tissue chronic inflammation [111,112]. From an anti-adipogenic and anti-lipotoxic perspective, tomato components limit adipocyte hypertrophy and ectopic lipid accumulation in the liver [113]. These effects are enhanced by activation of adiponectin signaling and AMPK phosphorylation mediated by lycopene and β-carotene, improving glucose uptake and insulin sensitivity [114]. As a mitochondrial protector, tomato antioxidants preserve organelle structural integrity by mitigating oxidative damage to DNA and proteins, thereby improving cellular metabolic efficiency [110]. Finally, its role as a gut microbiota modulator is attributed to its phenolic compounds and fiber, which promote microbial diversity and the production of anti-inflammatory metabolites [110]. Tomato consumption is associated with reduced weight gain and improved adiposity index in experimental models [111,113].

Nopal (*Opuntia ficus-indica*) has a complex bioactive profile integrating pigments (betalains, carotenoids), flavonoids such as isorhamnetin, and a significant dietary fiber fraction [56,115]. Its antioxidant activity extends beyond direct radical scavenging by inducing endogenous enzymes such as NRF2 and SOD-2, thereby mitigating oxidative damage in key metabolic organs [59,60]. Simultaneously, it exerts anti-inflammatory effects by inhibiting cytokines (IL-6, IL-8, TNF-α, IL-1β) and reducing macrophage infiltration in adipose tissue [60]. At the cellular level, nopal demonstrates anti-adipogenic and anti-lipotoxic properties; piscidic acid and isorhamnetin extracts reduce triglyceride accumulation and adipocyte size [58,116]. These actions, combined with reduced metabolic stress, contribute to its role as mitochondrial protector in metabolically active tissues [60]. As a modulator of the gut microbiota, its consumption increases beneficial genera (*Alloprevotella*, *Lachnospiraceae)* and upregulates occludin-1 expression, strengthening the intestinal barrier and reducing metabolic endotoxemia [61]. These mechanisms improve insulin sensitivity, reduce Homeostatic Model Assessment for Insulin Resistance (HOMA-IR), and reverse hepatic steatosis, establishing nopal as an integral dietary therapeutic strategy [60,116].

Quelites constitute a dense source of proteins, fiber, and essential minerals, notable for their richness in phenolic acids (chlorogenic, caffeic) and flavonoids such as rutin and quercetin [117]. Their high antioxidant capacity, validated through 2,2′-azino-bis(3-ethylbenzothiazoline-6-sulfonic acid (ABTS), DPPH, and ORAC assays, enables neutralization of free radicals and reduction in systemic oxidative stress, a critical factor in obesity progression [118,119]. Concurrently, they act as anti-inflammatory agents by modulating signaling pathways such as NF-κB and NLRP3, attenuating chronic low-grade inflammation [120]. Quelites also exhibit anti-adipogenic and anti-lipotoxic properties through downregulation of transcription factors such as PPARγ and C/EBPα, thereby inhibiting adipocyte differentiation and triglyceride accumulation [120]. Although direct evidence remains emerging, their flavonoid and carotenoid profile suggests a role as mitochondrial protectors by preserving energy efficiency under metabolic stress [119]. Finally, their fiber and phenolic content position them as relevant modulators of gut microbiota, promoting the growth of beneficial genera (*Lactobacillus*, *Bifidobacterium*) and SCFA production, thereby strengthening the intestinal barrier [120]. Collectively, their low energy density and high bioactive capacity promote adiposity reduction, improve insulin sensitivity, and optimize lipid profiles, consolidating them as a key ancestral resource for weight management [119,120].

The evidence demonstrates that the Milpa Diet constitutes a food system with exceptional antioxidant potential, derived from the phytochemical synergy among its components. Beyond serving as staple foods, these elements act as complementary sources of bioactive compounds, ranging from anthocyanins in purple maize to capsaicinoids in chili peppers and betalains in nopal. This bioactive matrix mitigates oxidative stress, counteracts lipotoxicity, and systemically modulates inflammatory processes.

This traditional dietary pattern therefore represents a high-value nutritional strategy with significant therapeutic potential for the prevention and management of obesity-related comorbidities. By optimizing mitochondrial function and acting as a robust modulator of gut microbiota, the Milpa system revalorizes a cultural legacy with critical applications in contemporary public health, offering a comprehensive approach to metabolic disorders.

## 5. Major Bioactive Compounds of the Milpa Diet and Their Role in Obesity

The Milpa Diet exhibits remarkable phytochemical diversity, with more than 34 bioactive compounds reported in the literature. For this analysis, a strategic selection of 20 metabolites was performed based on three fundamental criteria: (i) their significant quantitative presence in core components of Milpa system, (ii) robust mechanistic evidence supporting their direct antioxidant activity, and (iii) their documented role in modulating processes interconnected with oxidative stress in obesity, including inflammation, lipotoxicity, mitochondrial dysfunction, and gut microbiota modulation. This selection enables a focused examination of the most relevant phytochemicals without underestimating the complexity of the dietary system.

Obesity represents a complex pathophysiological state characterized by the expansion of adipose tissue, oxidative stress, chronic low-grade inflammation, mitochondrial dysfunction, and alterations in gut microbiota composition. These metabolic disturbances create a self-perpetuating cycle that sustains the disease and complicates its management. The Milpa Diet provides a food matrix with therapeutic potential. Its main components—maize, beans, chili peppers, squash, tomato, nopal, and quelites—are rich sources of bioactive compounds whose synergistic actions can modulate multiple pathways involved in obesity.

Focusing on the twenty compounds mentioned, their antioxidant, anti-inflammatory, anti-adipogenic, anti-lipotoxic, mitochondrial-protective, and gut microbiota-modulating effects have been analyzed. These compounds are:

− Flavonoids: Quercetin and Quercetin-3-glycoside [86,109,115,117,121,122,123,124,125,126,127,128,129,130,131,132,133]; Kaempferol and Kaempferol-3-O-glucoside [27,86,92,115,130,131,132,134,135,136,137,138,139,140,141,142,143,144,145]; Naringenin [86,92,109,131,132,133,145,146,147,148,149,150,151,152,153]; Anthocyanins [86,131,154,155,156,157,158,159,160,161,162,163,164]; Isorhamnetin and rhamnet-in-3-glucoside [115,165,166,167,168,169,170,171,172,173,174,175,176,177]; Rutin [109,115,117,128,129,177,178,179,180,181,182,183,184,185,186]; Catechin [128,129,133,172,173,174,175,176,178,179,180,181,182,183,184,185,186,187,188,189,190,191,192,193,194,195,196,197,198,199];
− Carotenoids: α-Carotene [99,103,109,200,201,202,203,204,205,206]; β-carotene [29,87,103,109,184,202,205,207,208,209,210,211,212,213,214,215,216,217,218,219]; Lycopene [103,109,111,212,220,221,222,223,224,225]; Lutein [87,103,109,219,226,227,228,229,230,231,232,233,234]; Zeaxanthin [87,103,219,235,236,237,238,239,240,241,242]; Capsanthin [102,103,108,243,244];
− Capsaicinoids: Capsaicin [50,102,104,186,245,246,247,248,249,250,251,252,253];
− Phenolic acids: Ferulic acid [109,115,117,130,218,219,221,254,255,256,257,258,259,260]; Chlorogenic acid [86,92,109,117,129,132,133,145,261,262,263,264,265]; Caffeic acid [86,92,109,117,130,133,145,186,219,266,267,268,269,270,271]; Gallic acid [29,86,92,115,117,133,145,186,262,272,273,274,275,276,277]; Betalains [234,278,279,280,281,282,283,284,285];
− Vitamins: γ-Tocoferol [66,115,286,287,288,289,290,291].

A summary of the structure, dietary source, biological activity, analytical methods, and molecular concentration of selected flavonoid compounds from the Milpa diet main foods is shown in Table 1.

Current evidence, illustrated in Supplemental Figures S1–S4, positions the Milpa Diet as a dietary model with potential applications for nutritional intervention in this condition.

## 6. Effects of Foods from the Milpa Diet and Their Bioactive Compounds on Obesity: In Vitro and In Vivo Evidence

The experimental validation of components of the Milpa Diet has demonstrated significant effects on body weight regulation and metabolic health through the six functional axes previously mentioned. In murine models [294], the impact of different maize tortilla variants in mice with obesity induced by a high-fat, high-fructose diet (HFFD) was evaluated. Among the interventions, which included conventional and blue maize as well as combinations with chia or amaranth, blue maize tortillas with beans stood out by inducing the lowest weight gain and a marked reduction in triglyceride levels.

**Figure 5:**
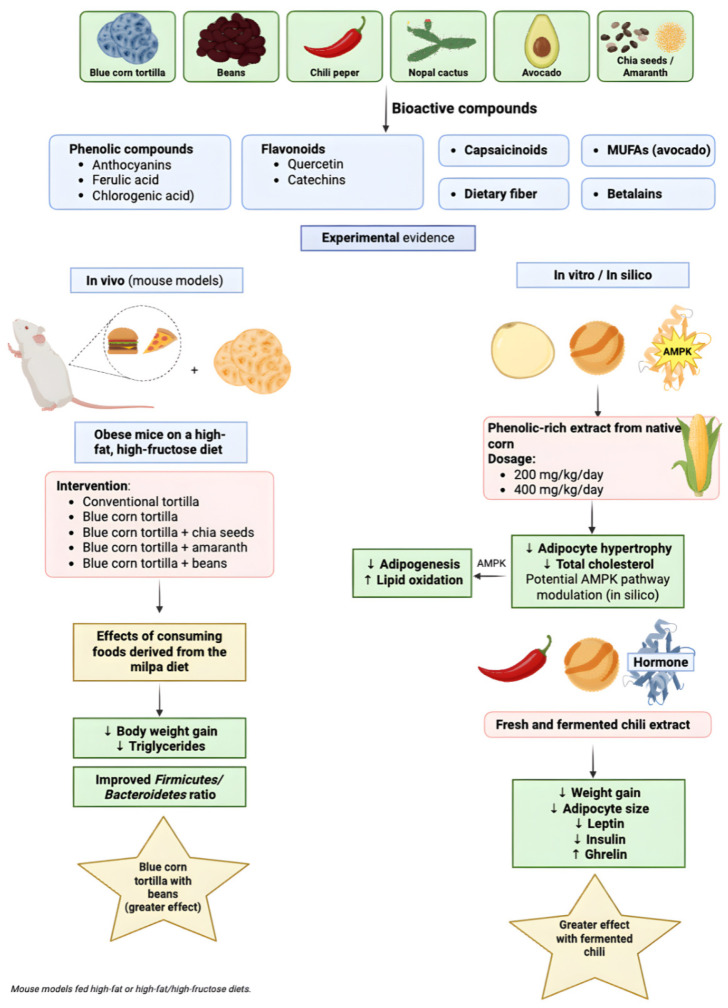
Foods of the Milpa Diet foods, their bioactive compounds, and anti-obesity effects: in vivo and in vitro evidence. Mouse models were fed high-fat or high-fat/high-fructose diets to induce obesity. In vitro and in silico approaches were used to investigate the potential molecular mechanisms of milpa-derived bioactive compounds, including AMPK pathway modulation. Arrow pointing up: Increase; Arrow pointing down: Decrease.

At intestinal ecosystem level, mice supplemented with these milpa-derived products exhibited substantial improvements in gastrointestinal microbiota. Compared with control groups, treated animals showed a favorable reconfiguration of the *Firmicutes/Bacteroidetes* ratio, suggesting that the combination of blue maize and legumes acts as a key factor in restoring intestinal eubiosis under obesogenic dietary conditions, as illustrated in Figure 5.

The evaluation of phenolic compounds from native maize has revealed significant anti-obesogenic potential through an integrated approach combining in silico analysis and in vivo models. Administration of maize extract—particularly at a dose of 400 mg/kg—in mice subjected to a high-fat diet (HFD) resulted in reduced total cholesterol levels and attenuation of adipocyte hypertrophy, outperforming lower-dose groups [295]. Complementarily, in silico computational analysis demonstrated that these secondary metabolites are capable of interacting with key proteins within the AMPK pathway. This finding suggests that native maize extract functions as a regulator of energy metabolism, promoting a protective profile against dysfunctional adipose tissue expansion and associated dyslipidemia [295].

The study of the anti-obesogenic effects of Capsicum (chili pepper) highlights the relevance of fermentation as a factor that enhances its metabolic properties. In murine models fed an HFD, administration of fermented chili extract (Group 4) demonstrated higher efficacy than the fresh form, resulting in the lowest body weight gain and preventing fat accumulation [236]. At the cellular and endocrine levels, the group supplemented with fermented chili exhibited the smallest adipocyte size among obesogenic diet groups. Simultaneously, this intervention improved the systemic hormonal profile by decreasing insulin and leptin levels while increasing ghrelin concentrations. These findings showed that the biotechnological processing of chili enhances its capacity to regulate the satiety–adiposity axis and mitigate dysfunctional expansion of adipose tissue [236].

Translational research in humans has validated the metabolic impact of traditional milpa-based food combinations. It also evaluated the glycemic response to a maize tortilla enriched with chia, nopal, flaxseed, and spinach in normal-weight individuals and those with overweight [296]. Following a crossover intervention, results demonstrated that consumption of this functional tortilla induced significantly lower glucose levels at two hours postprandial compared with both dextrose administration and conventional maize tortillas. These findings suggest that the integration of key milpa ingredients, such as nopal and chia, optimizes glycemic response in humans. This effect is likely attributable to the synergistic interaction between soluble fiber and bioactive compounds present in these foods, which act by modulating carbohydrate absorption and improving metabolic homeostasis [296]. Avocado is incorporated into the Milpa Diet as a key determinant of intestinal health. In a randomized clinical trial involving 163 adults [28], it was demonstrated that the daily inclusion of this fruit within an isocaloric diet significantly increases microbial diversity.

**Figure 6:**
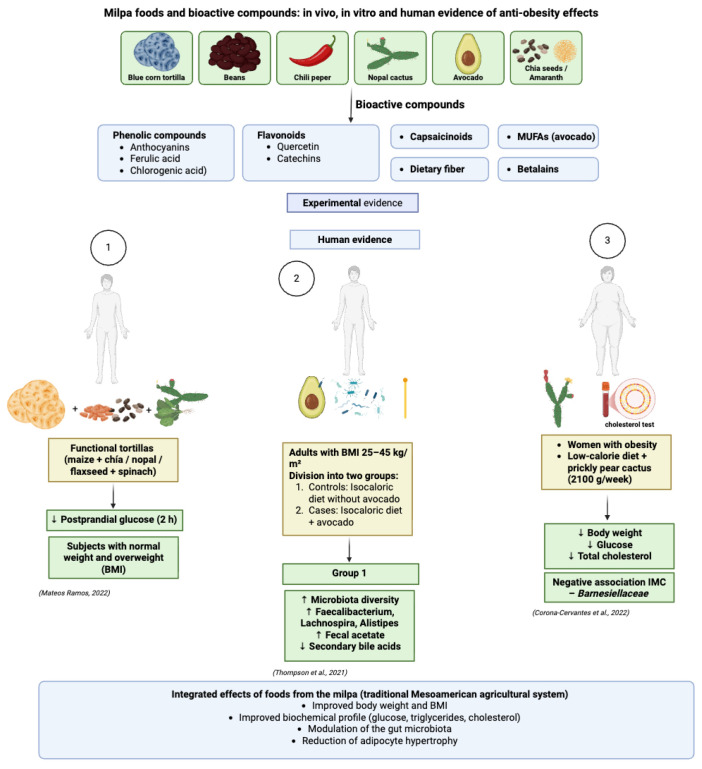
Human evidence of the metabolic effects of foods from the Milpa Diet system. Clinical and dietary intervention studies show that the consumption of milpa-derived foods, including functional maize tortillas enriched with chia, nopal, flaxseed and spinach, avocado, and nopal cactus, is associated with improvements in metabolic health. Reported outcomes include reduced postprandial glucose levels, decreased body weight, glucose and total cholesterol, as well as increased gut microbiota diversity and enrichment of beneficial bacterial taxa. These effects have been observed in adults with normal weight, overweight, and obesity, supporting the potential role of the milpa dietary pattern in obesity management and metabolic regulation. Arrow pointing up: Increase; Arrow pointing down: Decrease [28,296,297].

The intervention group exhibited enrichment of the genera *Faecalibacterium*, *Lachnospira*, and *Alistipes*, taxa associated with metabolic health. This taxonomic shift translated into a favorable alteration of the fecal metabolome: avocado consumers showed an 18% increase in acetate, a 70% increase in stearic acid, and a 98% increase in palmitic acid compared with controls. Concurrently, a reduction in cholic and chenodeoxycholic bile acids was observed, confirming that avocado exerts a regulatory effect on microbial metabolites and gastrointestinal function in humans [28]. The ability of nopal to improve metabolic health in the context of obesity was clinically validated. Women with obesity were evaluated undergoing an intervention of 2100 g/week of boiled nopal combined with a hypocaloric diet [297]. The results revealed reductions in body weight, glucose levels, and total cholesterol—effects not observed in the normal-weight control group—highlighting the efficacy of nopal as an adjunct to caloric restriction strategies. From microbiota perspective, the study identified a negative association between BMI and the abundance of the *Barnesiellaceae* family in both groups. This finding suggests that nopal consumption not only impacts biochemical parameters but also interacts with specific bacterial taxa linked to body weight regulation. The inclusion of nopal in a hypocaloric diet improves the metabolic profile of individuals with obesity [297], as illustrated in Figure 6.

## 7. Comparative Overview of the Milpa Diet, Mediterranean Diet, and DASH Diet

The Milpa Diet integrates health with traditional agricultural practices, distinguishing itself from global dietary patterns through its emphasis on crop regionality and seasonality [298,299]. The Mediterranean diet emerged in the mid-20th century following the “Seven Countries Study,” becoming a benchmark in cardiovascular disease prevention. This dietary pattern is characterized by high consumption of olive oil, whole grains, fatty fish, and red wine, conferring benefits such as reduced risk of type 2 diabetes and improved gut microbiota balance [300,301]. Meanwhile, the DASH diet (Dietary Approaches to Stop Hypertension) was developed in the 1990s with the specific aim of lowering blood pressure. Its nutritional framework prioritizes sodium reduction and increased intake of fiber, protein, and key minerals such as calcium, magnesium, and potassium, primarily through the consumption of low-fat dairy products, lean meats, and vegetables [77,302]. Despite their distinct origins, all three dietary models share fundamental pillars, including high consumption of fruits and vegetables, low sodium intake, and restriction of ultra-processed foods and red meat [77]. However, key differences remain: the Mediterranean diet includes moderate wine consumption, the DASH diet promotes dairy intake, and the Milpa Diet incorporates edible insects and uniquely emphasizes strict adherence to food seasonality and regionality [303].

An important aspect to highlight is that the Milpa Diet is rooted in the culinary traditions and ancestral knowledge of Mexico and Central America, while also accounting for food availability and regionality, making it an economically viable and culturally appropriate option for populations in these regions [304]. The DASH and, particularly, the Mediterranean diets may be less accessible for individuals in Mexico and Central America, as the cost of foods such as wine, nuts, and fatty fish can be relatively high in these settings, thereby limiting their widespread adoption [77]. Nevertheless, it is not appropriate to define a single dietary pattern as superior; rather, all of these models represent valid and health-promoting options. The selection of one dietary pattern over another should therefore be guided by individual factors, including taste preferences, economic capacity, food availability, and physical access. To summarize, key characteristics of the aforementioned dietary patterns are shown in Table 2.

### Strengths and Limitations

This review highlights the Milpa Diet as a traditional dietary system with significant biological and metabolic relevance, whose importance extends beyond its cultural roots to its potential role in metabolic health. The analysis of its core components—maize, beans, squash, chili peppers, tomatoes, nopal, and quelites—shows that this dietary pattern is inherently rich in bioactive compounds, including polyphenols, carotenoids, anthocyanins, and capsaicinoids, exhibiting antioxidant and anti-inflammatory properties. Accumulating evidence suggests that these compounds may modulate key processes involved in obesity-related metabolic dysfunction, such as oxidative stress, chronic low-grade inflammation, dysregulated adipogenesis, lipotoxicity, mitochondrial dysfunction, and alterations in the gut microbiota, in part through the regulation of cellular signaling pathways such as NF-κB and AMPK. Collectively, the available mechanistic and emerging experimental evidence supports the concept that the Milpa Diet represents a multiaxial dietary pattern with the potential to contribute to the prevention and attenuation of obesity-associated metabolic disturbances. Beyond its nutritional attributes, the Milpa system embodies principles of agroecological sustainability and biodiversity preservation, which may enhance its relevance within sustainable food and nutrition systems. From a public health perspective, the Milpa Diet may offer a culturally grounded and environmentally sustainable reference model for the development of dietary strategies aimed at improving metabolic health.

However, this review also shows several limitations. Despite promising mechanistic and preclinical findings, current clinical evidence evaluating this dietary pattern as a comprehensive intervention remains limited. Future research should prioritize the design of long-term, well-controlled clinical trials, complemented by advanced approaches such as metabolomics and nutrigenomics, to elucidate the interactions between Milpa-derived bioactive compounds, host metabolic regulation, and gut microbiota dynamics.

## 8. Evidence Interpretation and Limitations

An important distinction should be made between the evidence derived from individual foods or bioactive compounds and the evidence supporting the Milpa Diet as an integrated dietary pattern. In this review, the sections addressing the main foods of the Milpa Diet, including maize, beans, squash, chili pepper, tomato, nopal, and quelites, as well as their bioactive compounds, are primarily supported by mechanistic, analytical, preclinical, and emerging clinical evidence obtained from individual foods, food extracts, or isolated compounds. This level of evidence was used to describe plausible biological mechanisms involved in obesity-related metabolic dysfunction, including inflammation, oxidative stress, adipogenesis, lipotoxicity, mitochondrial function, and gut microbiota modulation.

By contrast, the evidence supporting the Milpa Diet as an integrated dietary pattern was interpreted separately and was based on studies evaluating traditional Mexican or Mesoamerican dietary patterns, dietary adherence, habitual food combinations, and their associations with obesity, body composition, cardiometabolic risk, or metabolic health outcomes. Therefore, the mechanistic effects reported for individual foods or isolated compounds should not be interpreted as direct clinical evidence of the complete Milpa Diet. Rather, they provide biological plausibility for the potential effects of this dietary model.

Accordingly, the Milpa Diet is presented in this review as a culturally relevant dietary pattern with mechanistic plausibility and potential metabolic benefits, supported by two complementary but distinct levels of evidence: mechanistic evidence from its component foods and bioactive compounds, and emerging epidemiological or clinical evidence on traditional dietary patterns. However, direct intervention studies evaluating the Milpa Diet as a complete dietary pattern remain limited. Future controlled clinical trials are needed to confirm whether the combined intake of these foods, within the traditional Milpa dietary model, produces additive or synergistic effects on obesity-related metabolic outcomes.

## 9. Conclusions

The reappraisal of the Milpa Diet as a traditional and biologically relevant dietary system underscores its potential contribution to sustainable and integrative approaches for addressing obesity and metabolic disorders.

Most mechanistic evidence of the Milpa Diet derives from individual foods or bioactive compounds, whereas clinical evidence stated the Milpa Diet as both a complete dietary pattern and, at same time, a culturally relevant Mexican dietary model plenty of mechanistic plausibility, and supported primarily by evidence from its component foods and bioactive compounds; however, this emerging evidence on traditional and healthy Mexican dietary patterns remains limited.

Overall, mechanistic and emerging clinical evidence suggests that the Milpa Diet represents a multi-axis nutritional strategy with potential to mitigate obesity-related metabolic dysfunction through coordinated effects on inflammation, oxidative stress, adipogenesis, lipotoxicity, mitochondrial function, and gut microbiota regulation. Although comprehensive clinical trials evaluating the Milpa Diet as an integrated intervention remain limited, current evidence supports its relevance for future translational research, public health strategies, and the development of sustainable dietary models aimed at improving metabolic health. Continued interdisciplinary research will be essential to validate its clinical applicability and to define its role in evidence-based nutritional recommendations.

## Figures and Tables

**Table 1:** Anti-obesity effects of bioactive compounds from the main foods of the Milpa Diet.

Compounds	Structure	Dietary Source	Biological Activity	Analytical Methods	Molecular Concentration
**Flavonoids**					
Quercetin (aglycone) Quercetin-3-glucoside (glycosylated)	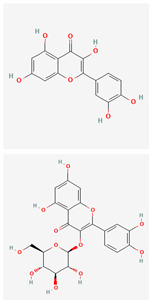	Maize (M) (*Zea mays* L.) [86,130] Common bean (B) (*Phaseolus vulgaris* L.) [131] Chili pepper (Ch) (*Capsicum annuum* L.) [132] Tomato (T) (*Solanum lycopersicum* L.) [109,133] Nopal cactus (N) (*Opuntia ficus-indica*) [115] Quelites (Q) (wild edible greens) [117]	Antioxidant; anti-inflammatory; anti-adipogenic; anti-lipotoxic; mitochondrial-protective; gut microbiota-modulator [121,122,123,124,125,126,127,128,129].	HPLC–QTOF-MS (M)(CH) [86,132] HPLC-DAD/ESI-MS/MS (M) [130] HPLC-PDA (F) [131] HPLC (T) [109,115] HPLC-DAD-FLD (T) [133] HPLC-DAD (Q) [117]	Glycosylated: 14.7 ± 0.6 (μg/g) (M) [86] 3.9–15.2 (mg/L) (F) [131] 14.7 ± 0.6 (μg/g) (CH) [132] Aglycone: 7.6–99.0 (μg/g) (M) [130] 0.7–4.4 (mg/100 g) (T) [109] 5.55 (μg/g) (T) [133] 4.32 (mg/100 g) (N) [115] 135.13 ± 0.01 (μg/g) (Q) [117]
Kaempferol (aglycone) Astragalin or Kaempferol-3-O-glucoside (glycosylated)	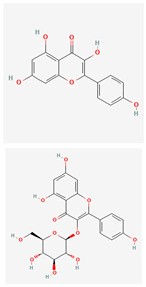	Maize (M)(*Zea mays* L.) [96,130] Common bean (F) (*Phaseolus vulgaris* L.) [92,131,145] Chili pepper (CH) (*Capsicum annuum* L.) [132] Nopal cactus (N) (*Opuntia ficus-indica*) [115]	Antioxidant; anti-inflammatory; anti-adipogenic; anti-lipotoxic; mitochondrial-protective; gut microbiota-modulator [27,134,135,136,137,138,139,140,141,142,143,144].	HPLC–QTOF-MS (M) [86] HPLC-PDA (F) [92,145] HPLC-DAD/ESI-MS/MS (M) [130] HPLC (N) [115]	Glycosylated: 25.8 ± 1.5 (μg/g) (M) [86] 1.5–1.9 (mg/g) (F) [92,145] 25.3–39.5 (mg/L) (F) [131] 25.8 ± 1.5 (μg/g) (CH) [132] Aglycone: 11.9–44.0 (μg/g) (M) [130] 0.22–2.7 (mg/100 g) (N) [115]
Naringenin	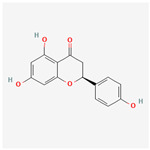	Maize (M) (*Zea mays* L.) [86] Common bean (F) (*Phaseolus vulgaris* L.) [92,131,145] Chili pepper (CH) (*Capsicum annuum* L.) [132] Tomato (T) (*Solanum lycopersicum* L.) [109,133]	Antioxidant; anti-inflammatory; anti-adipogenic; anti-lipotoxic; mitochondrial-protective; gut microbiota-modulator [146,147,148,149,150,151,152,153].	HPLC–QTOF-MS (M) (CH) [86,132] HPLC-PDA (F) [92,131,145] HPLC (T) [109] HPLC-DAD-FLD (T) [133]	2.0 ± 0.1 (μg/g) (M) [86] 1.9–2.1 (mg/g) (F) [92,145] 1.9–2.1 (mg/L) (F) [131] 2.0 ± 0.1 (μg/g) (CH) [132] 0–1.3 (mg/100 g) (T) [109] 2.98 (μg/g) (T) [133]
Anthocyanins	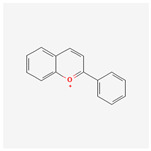	Maize (M) (*Zea mays* L.) [86] Common bean (F) (*Phaseolus vulgaris* L.) [131] Squash (CA) (*Cucurbita pepo* L.) [164]	Antioxidant; anti-inflammatory; anti-adipogenic; anti-lipotoxic; mitochondrial-protective; gut microbiota-modulator [154,155,156,157,158,159,160,161,162,163].	HPLC–QTOF-MS (M) [86] pH differential (F) (CA) [131,132,133,145,164]	1460.4 (μg/g) (M) [86] 10.7–66.3 (mg C3GE/L) (F) [131] 0.018–0.098 (CA) (mg/100 g) [164]
Isorhamnetin (aglycone) Isorhamnetin-3-glucoside (glycosylated)	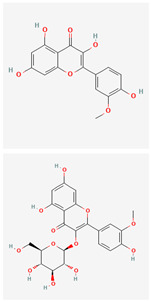	Maize (M) (*Phaseolus vulgaris* L.) [177] Nopal (N) (*Opuntia ficus-indica*) [115]	Antioxidant; anti-inflammatory; anti-adipogenic; anti-lipotoxic; mitochondrial-protective; gut microbiota-modulator [165,166,167,168,169,170,171,172,173,174,175,176].	HPLC-MS (F) [177] HPLC (N) [115]	Glycosylated: 0.40–0.51 (mg/g) (F) [177] Aglycone: 2.41–91 (mg/100 g) (N) [115]
Rutin	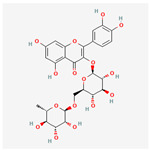	Common bean (F) (*Phaseolus vulgaris* L.) [177] Chili pepper (CH) (*Capsicum annuum* L.) [186] Tomato (T) (*Solanum lycopersicum* L.) [109] Nopal cactus (N) (*Opuntia ficus-indica*) [115] Quelites (Q) [117]	Antioxidant; anti-inflammatory; anti-adipogenic; anti-lipotoxic; mitochondrial-protective; gut microbiota-modulator [128,129,178,179,180,181,182,183,184,185].	HPLC-MS (F) [177] HPLC-UV (CH) [186] HPLC (T) (N) [109,115] HPLC-DAD (Q) [117]	1.15–1.25 (mg/g) (F) [177] 0.20–7.90 (mg/100 g PF) (CH) [186] 0.5–4.5 (mg/100 g) (T) [109] 2.36–26.17 (mg/100 g) (N) [115] 2683.14 ± 0.50 (μg/g) (Q) [117]
Catechin	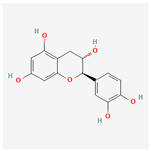	Squash (CA) (*Cucurbita pepo* L.) [199] Chili pepper (CH) (*Capsicum annuum* L.) [186] Tomato (T) (*Solanum lycopersicum* L.) [133]	Antioxidant; anti-inflammatory; anti-adipogenic; anti-lipotoxic; mitochondrial-protective; gut microbiota-modulator [128,129,172,173,174,175,176,178,179,180,181,182,183,184,185,187,188,189,190,191,192,193,194,195,196,197,198].	HPLC/UV-VIS (CA) [199] HPLC-UV (CH) [186] HPLC-DAD-FLD (T) [133]	0.11–0.31 (mg/100 g PF) (CA) [199] 0.11–3.68 (mg/100 g PF) (CH) [186] 260.50 (μg/g) (T) [133]
**Carotenoids**					
α-carotene	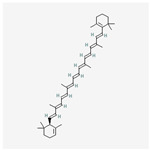	Squash (CA) (*Cucurbita pepo* L.) [99,206] Chili pepper (CH) (*Capsicum annuum* L.) [103] Tomato (T) (*Solanum lycopersicum* L.) [109]	Antioxidant; anti-inflammatory; anti-adipogenic; anti-lipotoxic; mitochondrial-protective; gut microbiota-modulator [200,201,202,203,204,205].	HPLC-PDA (CA) [206] HPLC-UV/DAD (CA) [99] HPLC (CH) (T) [103,109]	Not quantified (CA) [206] 0.1–39.48 (µg/g) (CA) [99] 0.79 (mg/100 g) (CH) [103] 0–0.002 (mg/100 g PF) (T) [109]
β-carotene	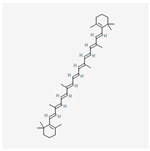	Maize (M) (*Zea mays* L.) [29,87] Squash (CA) (*Cucurbita pepo* L.) [219] Chili pepper (CH) (*Capsicum annuum* L.) [103] Tomato (T) (*Solanum lycopersicum* L.) [109]	Antioxidant; anti-inflammatory; anti-adipogenic; anti-lipotoxic; mitochondrial-protective; gut microbiota-modulator [184,202,205,207,208,209,210,211,212,213,214,215,216,217].	HPLC-DAD (M) [87,218] HPLC-DAD-ESI/MS/MS (CA) [219] HPLC/Spectrophotometry (CH) [103] HPLC (T) [109]	0.21–2.42 mg/kg (M) [218] 0.16–7.24 (µg/g) (M) [87] 0.54–0.60 (mg/100 g) (CA) [219] 0.15–28.39 (mg/100 g) (CH) [103] 0.1–1.2 (mg/100 g PF) (T) [109]
Lycopene	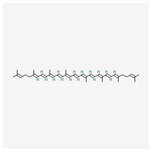	Chili pepper (CH) (*Capsicum annuum* L.) [103] Tomato (T) (*Solanum lycopersicum* L.) [109]	Antioxidant; anti-inflammatory; anti-adipogenic; anti-lipotoxic; mitochondrial-protective; gut microbiota-modulator [111,212,220,221,222,223,224,225].	Not specified (CH) [103] HPLC (T) [109]	4.69 (mg/g) (CH) [103] 7.8–18.1 (mg/100 g PF) (T) [109]
Lutein	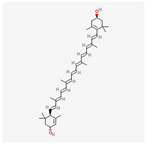	Maize (M) (*Zea mays* L.) [87] Squash (CA) (*Cucurbita pepo* L.) [219] Chili pepper (CH) (*Capsicum annuum* L.) [103] Tomato (T) (*Solanum lycopersicum* L.) [109] Nopal cactus (N) (*Opuntia ficus-indica*) [234]	Antioxidant; anti-inflammatory; anti-adipogenic; anti-lipotoxic; mitochondrial-protective [226,227,228,229,230,231,232,233].	HPLC-DAD (M) [87] HPLC-DAD-ESI/MS/MS (CA) [219] HPLC (CH) (T) [103,109] Not specified (N) [234]	0.16–21.17 µg/g (M) [87] 0.02–0.03 (CA) (mg/100 g) [219] 8.75–21.08 (CH) (mg/100 g) [103] 0.09 (mg/100 g PF) (T) [109] 10.03–21.10 (µg/g) (N) [234]
Zeaxanthin	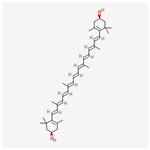	Maize (M) (*Zea mays* L.) [87] Squash (CA) (*Cucurbita pepo* L.) [219] Chili pepper (CH) (*Capsicum annuum* L.) [103]	Antioxidant; anti-inflammatory; anti-adipogenic; anti-lipotoxic; mitochondrial-protective; gut microbiota-modulator [235,236,237,238,239,240,241,242].	HPLC-DAD (M) [87] HPLC-DAD-ESI/MS/MS (CA) [219] HPLC (CH) [103]	0.08–10.71 µg/g (M) [87] 2.65–2.91 (mg/100 g) (CA) [219] (0.63–151.39 mg/100 g) (CH) [103]
Capsanthin	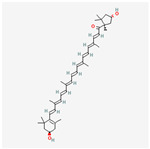	Chili pepper (*Capsicum annuum* L.) (CH) [102,103]	Antioxidant; anti-inflammatory; anti-adipogenic; anti-lipotoxic; gut microbiota-modulator [108,243,244,292,293].	HPLC/Spectrophotometry (CH) [103] HPLC (CH) [102]	31.8–4442 (mg/100 g) (CH) [103] 6.97 (mg/100 g de DW) (CH) [102]
**Capsaicinoids**					
Capsaicin		Chili pepper (*Capsicum annuum* L.) [102,186,252,253]	Antioxidant; anti-inflammatory; anti-adipogenic; anti-lipotoxic; mitochondrial-protective; gut microbiota-modulator [50,104,245,246,247,248,249,250,251].	HPLC-GC-MS (CH) [102] HPLC-UV (CH) [252,253] UHPLC-PDA/ESI-MS (CH) [186]	600–13,000 (ppm) (CH) [102] 129–3352 (ppm) (CH) [253] 0.44–0.53 (hot); 0.03–0.05 (semi-hot varieties) (mg/g) (CH) [252] 28.23–2322.35 (µg/g) (CH) [186]
**Phenolic acids**					
Ferulic acid	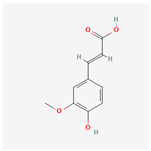	Maize (M) (*Zea mays* L.) [130,218] Squash (CA) (*Cucurbita pepo* L.) [219] Tomato (T) (*Solanum lycopersicum* L.) [109] Nopal cactus (N) (*Opuntia ficus-indica*) [115] Quelites (Q) [117]	Antioxidant; anti-inflammatory; anti-adipogenic; anti-lipotoxic; mitochondrial-protective; gut microbiota-modulator [221,254,255,256,257,258,259,260].	HPLC-UV (M) [218] HPLC-DAD/ESI-MS/MS (M) [130] HPLC-MS/MS (CA) [219] HPLC (T) (N) [109,115] HPLC-DAD (Q) [117]	1556.24–4521.26 (µg/g) (M) [218] 3695–5991 (μg/g) (M) [130] 4.72–5.17 (mg/100 g) (CA) [219] 0.2–0.5 (mg/100 g PF) (T) [109] 0.56–34.77 (mg/100 g) (N) [115] 316.95 ± 0.40 (µg/g) (Q) [117]
Chlorogenic acid	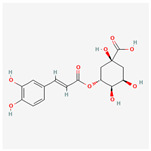	Maize (M) (*Zea mays* L.) [86] Common bean (F) (*Phaseolus vulgaris* L.) [92,145] Chili pepper (CH) (*Capsicum annuum* L.) [132] Tomato (T) (*Solanum lycopersicum* L.) [109,133] Quelites (Q) [117]	Antioxidant; anti-inflammatory; antiadipogenic; anti-lipotoxic; mitochondrial-protective; gut microbiota-modulator [129,261,262,263,264,265].	HPLC–QTOF-MS (M) [86] HPLC-PDA (F) [92,145] HPLC-UV (CH) [132] HPLC (T) [198] HPLC-DAD-FLD (T) [133] HPLC-DAD (Q) [117]	6.6 ± 0.5 (μg/g) (M) [86] 1.1–3.6 (mg/g) (F) [92,145] 0.20–1.79 (CH) (mg/100 g PF) [132] 1.4–3.3 (mg/100 g PF) (T) [109] 1411.59 (μg/g) (T) [133] 270.82 ± 0.07 (μg/g) (Q) [117]
Caffeic acid	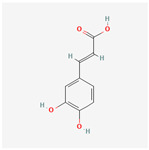	Maize (M) (*Zea mays* L.) [86,130] Common bean (F) (*Phaseolus vulgaris* L.) [92,145] Squash (CA) (*Cucurbita pepo* L.) [219] Chili pepper (CH) (*Capsicum annuum* L.) [186] Tomato (T) (*Solanum lycopersicum* L.) [109,133] Quelites (Q) [117]	Antioxidant; anti-inflammatory; anti-adipogenic; anti-lipotoxic; mitochondrial-protective; gut microbiota-modulator [266,267,268,269,270,271].	HPLC–QTOF-MS (M) [86] HPLC-DAD (M) (F) (Q) [92,117,130,145] HPLC-MS/MS (CA) [219] HPLC-UV (CH) [186] HPLC (T) [109] HPLC-DAD-FLD (T) [133]	1296.8 ± 103.7 (μg/g) (M) [86] 93–350 (μg/g) (M) [130] 0.9–3.3 (mg/g) (F) [92,145] 3.41–3.83 (mg/100 g) (CA) [219] 0.20–2.20 (mg/100 g PF) (CH) [186] 0.1–1.3 (mg/100 g PF) (T) [109] 37.72 (μg/g) (T) [133] 130.71 ± 0.05 (μg/g) (Q) [117]
Gallic acid	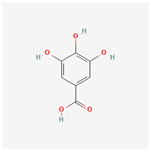	Maize (M) (*Zea mays* L.) [29,86] Common bean (F) (*Phaseolus vulgaris* L.) [92,145] Chili pepper (CH) (*Capsicum annuum* L.) [186] Tomato (T) (*Solanum lycopersicum* L.) [133] Nopal cactus (N) (*Opuntia ficus-indica*) [115] Quelites (Q) [117]	Antioxidant; anti-inflammatory; antiadipogenic; anti- lipotoxic; mitochondrial-protective; gut microbiota-modulator [262,272,273,274,275,276,277].	HPLC–QTOF-MS (M) [86] HPLC-PDA (F) [92,145] HPLC-UV (CH) [186] HPLC-DAD-FLD (T) [133] HPLC (N) [115] HPLC-DAD (Q) [117]	1.3 ± 0.1 (μg/g) (M) [86] 8.2 ± 2.8 (mg/g) (F) [92,145] 49.10–101.30 (mg/100 g PF) (CH) [186] 99.29 (μg/g) (T) [133] 0.64–2.37 (mg/100 g) (N) [115] 45.04 ± 0.06 (μg/g) (Q) [117]
Betalains	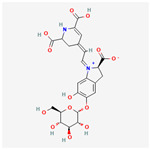	Nopal cactus (N) (*Opuntia ficus-indica*) [234,285]	Antioxidant; anti-inflammatory; anti-adipogenic; anti-lipotoxic; mitochondrial-protective; gut microbiota-modulator [278,279,280,281,282,283,284].	HPLC-DAD-ESI-MS (N) [285] HPLC(N) [234]	0.21–1.16 (mg/g PS) (N) [285] 40.6 (mg/100 g) (N) [234]
**Vitamins**					
γ-tocoferol	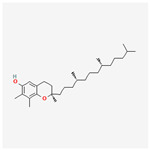	Squash (CA) (*Cucurbita pepo* L.) [291] Nopal cactus (N) (*Opuntia ficus-indica*) [115]	Antioxidant; anti-inflammatory; anti-adipogenic; anti-lipotoxic; mitochondrial-protective; gut microbiota-modulator [66,286,287,288,289,290].	HPLC–fluorescence detection (CA) [291] HPLC (N) [115]	36.0–352.4 (mg/kg lipid fraction) (CA) [291] 7.9–174 (mg/100 g) (N) [115]

**CA:** Squash; **CH**: Chili pepper; **DW:** Dry Weight; **F:** Common Bean; **FW:** Fresh weight; **M:** Maize; **N:** Nopal; **Q:** Quelite; **T:** Tomato.

**Table 2:** Comparative Overview of *the Milpa Diet*, Mediterranean Diet, and DASH Diet.

Diet	Main Characteristics	Key Nutrients	Main Foods
**Milpa**	Dietary pattern based on foods cultivated within the milpa system.	Potassium, dietary fiber, and phosphorus.	Maize, beans, squash, and chili peppers, along with regional and seasonal foods.
**Mediterranean**	Traditional dietary pattern of Mediterranean countries.	Polyunsaturated and monounsaturated fatty acids, potassium, and dietary fiber	Fruits, vegetables, olive oil, fatty fish, nuts, and dried fruits
**DASH**	Dietary pattern designed to reduce sodium intake and blood pressure.	Potassium, magnesium, calcium, and dietary fiber	Fruits, vegetables, lean meats, and dairy products

## Data Availability

Requestors wishing to access the data used in this study can make a request to pep.tur@uib.es.

## Supplementary Materials

The following supporting information can be downloaded at https://www.mdpi.com/article/10.3390/nu18121991/s1, Supplementary Table S1. Search strategies, search strings, and Boolean operators used for each database. Supplementary Figure S1. Antioxidant and anti-inflammatory effects of bioactive compounds derived from the Milpa Diet in obesity. Supplementary Figure S2. Antiadipogenic and Anti-Lipotoxic Effects of Bioactive Compounds from Functional Foods of the Milpa Diet. Supplementary Figure S3. Mitochondrial dysfunction and protection mechanisms mediated by bioactive compounds derived from the Milpa Diet in adipose tissue. Supplementary Figure S4. Modulation of Gut Microbiota and Intestinal Barrier Function by Bioactive Compounds from the Milpa Diet.
