# Supplementary material for: Multi-Axis Functional Mechanisms of the Milpa Diet in Obesity: A Scoping Review
Source: Nutrients. 2026 Jun 19;18(12):1991. doi: 10.3390/nu18121991 (PMC13306012; doi:10.3390/nu18121991)
Supplement: Supplementary file 1 [file nutrients-18-01991-s001.zip › nutrients-4345812-supplementary.pdf]

Supplementary Table S1. Search strategies, search strings, and Boolean operators used for each database.

Database	Search date	Search string	Filters applied	Records
Medline via PubMed	May 2026	(“Milpa Diet” OR “traditional Mexican diet” OR “maize” OR “corn” OR “Zea mays” OR “bean” OR “Phaseolus vulgaris” OR “squash” OR “pumpkin” OR “Cucurbita pepo” OR “chili” OR “Capsicum annuum” OR “tomato” OR “Solanum lycopersicum” OR “nopal” OR “Opuntia ficus-indica” OR quelites) AND (“obesity” OR “metabolic dysfunction” OR “insulin resistance” OR “inflammation” OR “oxidative stress” OR “adipogenesis” OR “lipotoxicity” OR “mitochondrial dysfunction” OR “gut microbiota”) AND (“bioactive compounds” OR “polyphenols” OR “flavonoids” OR “carotenoids” OR “capsaicinoids” OR “phenolic acids” OR “betalains” OR “tocopherols”)	English OR Spanish; peer-reviewed articles; human, animal, in vitro, clinical trial, and randomized controlled trial studies when applicable	Included in final pool
Scopus	May 2026	TITLE-ABS-KEY (“Milpa Diet” OR “traditional Mexican diet” OR “maize” OR “corn” OR “Zea mays” OR “bean” OR “Phaseolus vulgaris” OR “squash” OR “pumpkin” OR “Cucurbita pepo” OR “chili” OR “Capsicum annuum” OR “tomato” OR “Solanum lycopersicum” OR “nopal” OR “Opuntia ficus-indica” OR “quelites”) AND TITLE-ABS-KEY (“obesity” OR “metabolic dysfunction” OR “insulin resistance” OR “inflammation” OR “oxidative stress” OR “adipogenesis” OR “lipotoxicity” OR “mitochondrial dysfunction” OR “gut microbiota”) AND TITLE-ABS-KEY (“bioactive compounds” OR	Article; English OR Spanish; human, animal, in vitro, clinical trial, and randomized controlled trial studies when applicable	Included in final pool

		“polyphenols” OR “flavonoids” OR “carotenoids” OR “capsaicinoids” OR “phenolic acids” OR “betalains” OR “tocopherols”)		
Web of Science	May 2026	TS=(“Milpa Diet” OR “traditional Mexican diet” OR maize OR corn OR “Zea mays” OR bean OR “Phaseolus vulgaris” OR squash OR pumpkin OR “Cucurbita pepo” OR chili OR “Capsicum annuum” OR tomato OR “Solanum lycopersicum” OR nopal OR “Opuntia ficus-indica” OR quelites) AND TS=(obesity OR “metabolic dysfunction” OR “insulin resistance” OR “inflammation” OR “oxidative stress” OR “adipogenesis” OR “lipotoxicity” OR “mitochondrial dysfunction” OR “gut microbiota”) AND TS=(“bioactive compounds” OR “polyphenols” OR “flavonoids” OR “carotenoids” OR “capsaicinoids” OR “phenolic acids” OR “betalains” OR “tocopherols”)	Article; English OR Spanish; human, in vitro, clinical trial, and randomized controlled trial studies when applicable	Included in final pool
Final pool	May 2026	Records from Medline via PubMed, Scopus, and Web of Science were merged and duplicates were removed before screening.	Eligibility criteria described in Methods	344 records screened; 40 records excluded; 304 records included
ChEMBL	May 2026	Selected compounds were searched individually by compound name: quercetin, kaempferol, naringenin, anthocyanins, isorhamnetin, rutin, catechin, α-carotene, β-carotene, lycopene, lutein, zeaxanthin, capsanthin, capsaicin, ferulic acid, chlorogenic acid, caffeic acid, gallic acid, betalains, and γ-tocopherol.	Used only for verification of chemical structures and biochemical annotations; not used for bibliographic study identification	Not applicable

Supplementary Figures

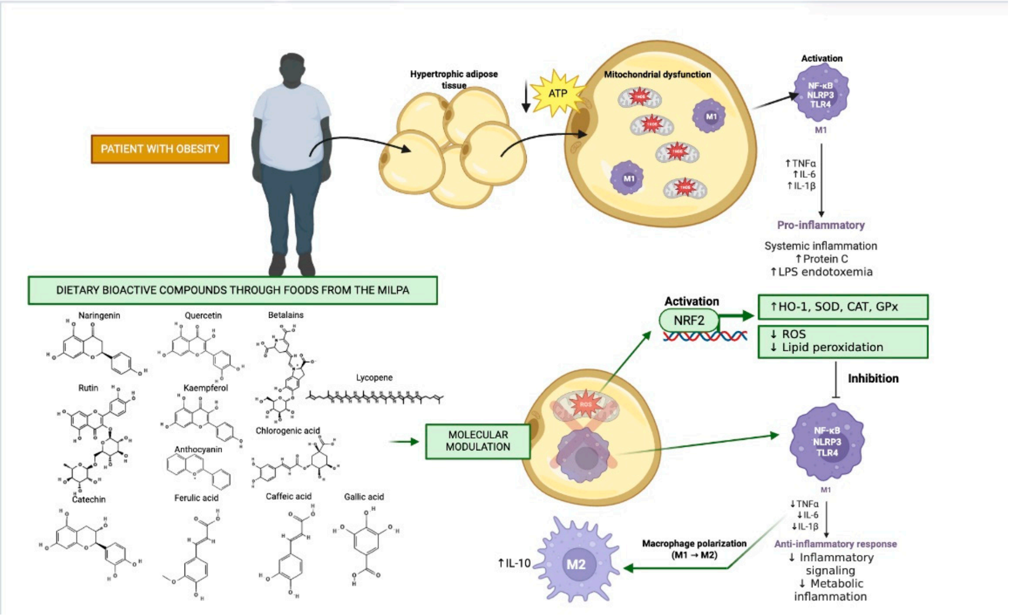

Supplementary Figure S1. Antioxidant and anti-inflammatory effects of bioactive compounds derived from *the Milpa Diet* in obesity. In hypertrophic adipose tissue, mitochondrial dysfunction and increased reactive oxygen species (ROS) activate pro-inflammatory signaling pathways, including NF-κB, NLRP3, and TLR4, promoting macrophage M1 polarization and systemic inflammation. Dietary bioactive compounds such as flavonoids, phenolic acids, carotenoids, and betalains activate the Nrf2 pathway, enhancing endogenous antioxidant defenses (HO-1, SOD, CAT, GPx), reducing ROS production and lipid peroxidation. These effects inhibit pro-inflammatory signaling and favor macrophage polarization toward the anti-inflammatory M2 phenotype, contributing to improved adipose tissue function and reduced metabolic inflammation.

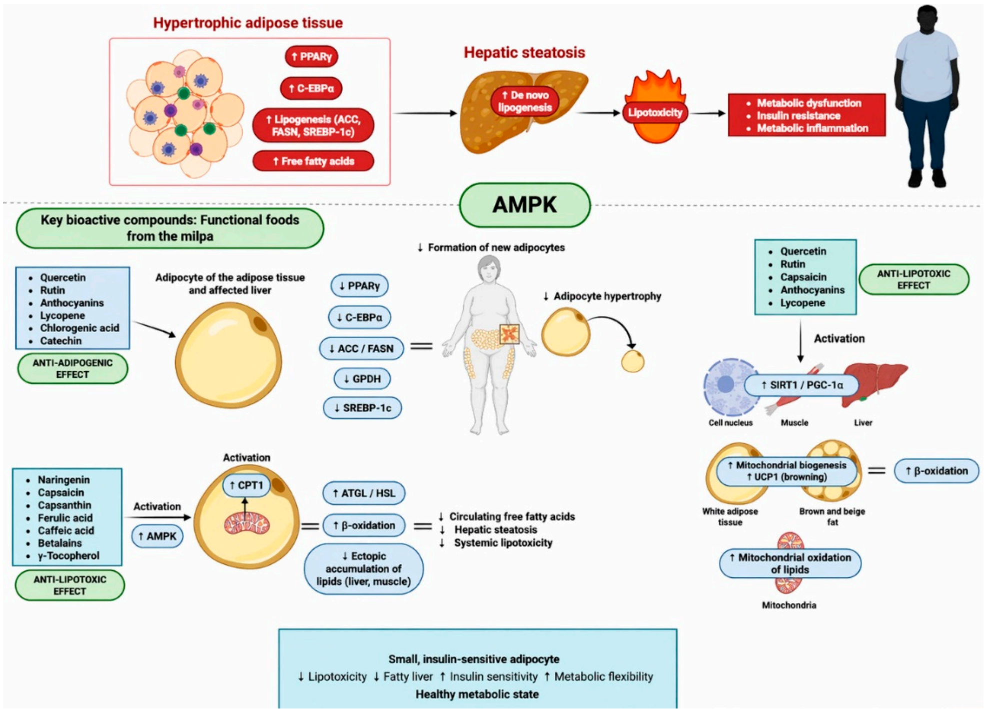

Supplementary Figure S2. Antiadipogenic and Anti-Lipotoxic Effects of Bioactive Compounds from Functional Foods of *the Milpa Diet*. Obesity is characterized by hypertrophic adipose tissue, increased adipogenesis and lipogenesis, elevated circulating free fatty acids, and ectopic lipid accumulation, leading to hepatic steatosis, lipotoxicity, insulin resistance, and metabolic inflammation. Bioactive compounds derived from functional foods of *the Milpa Diet*, including quercetin, rutin, anthocyanins, naringenin, capsaicin, lycopene, chlorogenic acid, and betalains, exert potent antiadipogenic and anti-lipotoxic effects through the activation of AMP-activated protein kinase (AMPK). These compounds suppress adipocyte differentiation and hypertrophy by downregulating key adipogenic and lipogenic transcription factors (PPARγ, C/EBPα, SREBP-1c) and enzymes (ACC, FASN), while simultaneously enhancing lipid catabolism via the upregulation of CPT1, ATGL, and HSL. AMPK activation further promotes mitochondrial biogenesis and fatty acid oxidation through the SIRT1/PGC-1α axis, inducing browning of white adipose tissue and increasing β-oxidation. Collectively, these mechanisms reduce circulating free fatty acids, prevent ectopic lipid deposition, alleviate hepatic steatosis, and may contribute to improving insulin sensitivity, leading to a small, insulin-sensitive adipocyte phenotype and improved metabolic homeostasis.

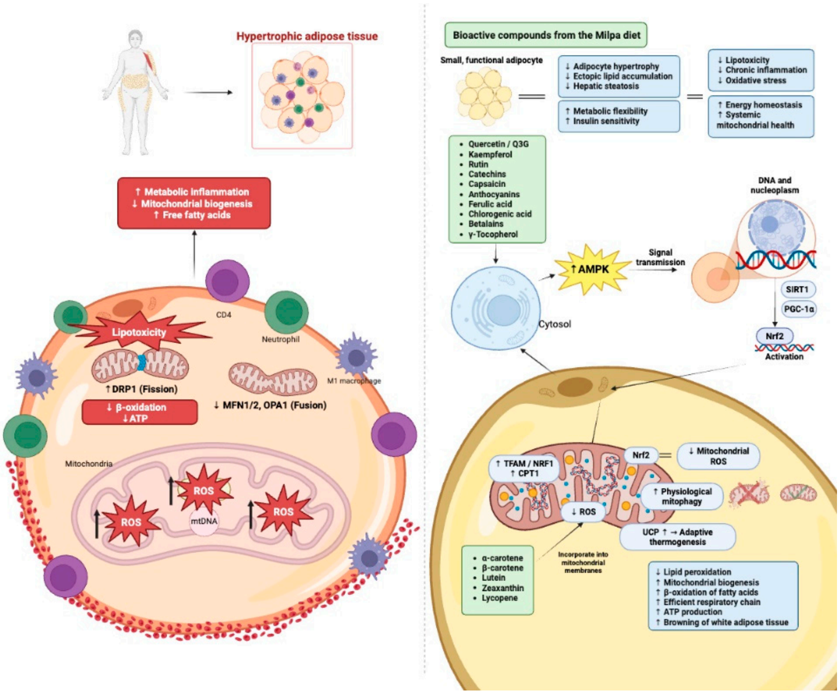

Supplementary Figure S3. Mitochondrial dysfunction and protection mechanisms mediated by bioactive compounds derived from *the Milpa Diet* in adipose tissue. Obesogenic conditions promote adipose tissue hypertrophy, mitochondrial dysfunction, lipotoxicity, oxidative stress, and chronic inflammation, characterized by impaired mitochondrial dynamics, reduced fatty acid β-oxidation, and decreased ATP production. In contrast, bioactive compounds derived from *the Milpa Diet*—particularly flavonoids, phenolic acids, carotenoids, and tocopherols—activate key metabolic regulators such as AMPK, SIRT1/PGC-1α, and NRF2. These pathways enhance mitochondrial biogenesis, physiological mitophagy, antioxidant defenses, and UCP-mediated adaptive thermogenesis, leading to improved mitochondrial efficiency, reduced oxidative damage, and the maintenance of small, functional adipocytes and systemic metabolic homeostasis.

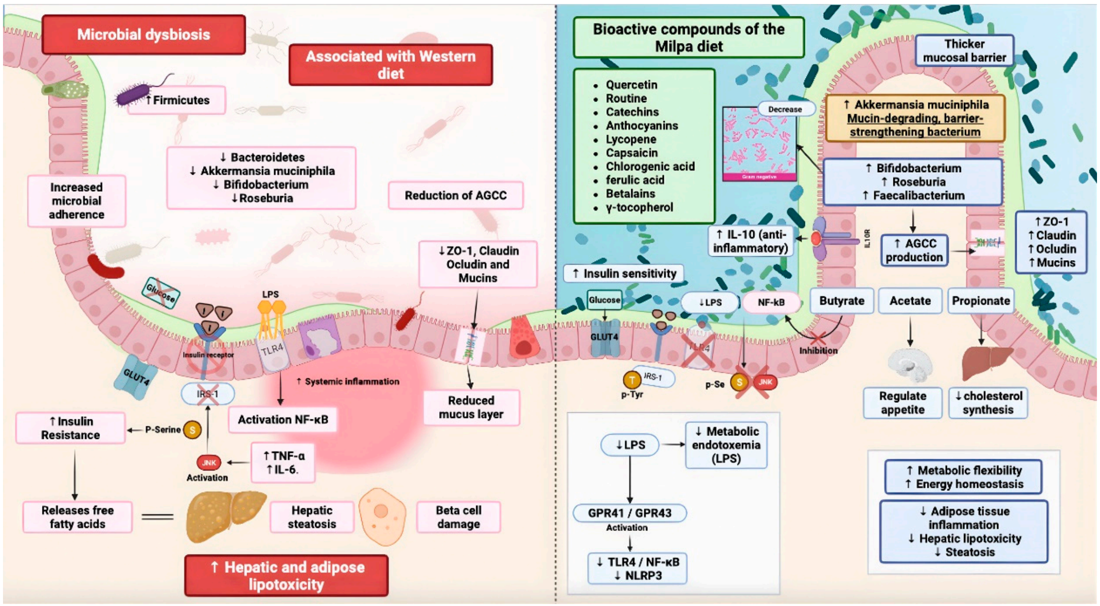

Supplementary Figure S4. Modulation of Gut Microbiota and Intestinal Barrier Function by Bioactive Compounds from *the Milpa Diet*. Bioactive compounds from *the Milpa Diet*, including flavonoids (quercetin, rutin, catechins, anthocyanins), phenolic acids (chlorogenic and ferulic acids), carotenoids (lycopene), capsaicinoids, betalains, and γ-tocopherol, contribute to the modulation of gut microbiota composition and intestinal barrier integrity. These compounds promote the enrichment of beneficial bacteria such as *Akkermansia muciniphila, Bifidobacterium, Roseburia*, and *Faecalibacterium*, enhancing short-chain fatty acid (SCFA) production (butyrate, acetate, and propionate). Increased SCFA availability activates GPR41/GPR43 signaling, reduces lipopolysaccharide (LPS)-induced metabolic endotoxemia, and attenuates TLR4/NF-κB/NLRP3–mediated inflammatory pathways. Concurrently, bioactive compounds strengthen tight junction proteins (ZO-1, claudin, occludin) and mucin production, improving intestinal barrier function. Collectively, these mechanisms lead to improved insulin sensitivity, reduced systemic inflammation, and decreased hepatic and adipose tissue lipotoxicity.
